# The Pregnane-X receptor regulates steroid synthesis in mouse Leydig cells

**DOI:** 10.3389/fendo.2024.1430781

**Published:** 2025-05-06

**Authors:** Emmanuelle Martinot, Hélène Holota, Angélique de Haze, Claude Beaudoin, David H. Volle

**Affiliations:** Institut national de la santé et de la recherche médicale (INSERM) U1103, Université Clermont Auvergne, Centre national de la recherche scientifique (CNRS) UMR-6293, GReD Institute, Team-Volle, Clermont–Ferrand, France

**Keywords:** PXR, testosterone, Leydig cells, mouse, xenobiotics

## Abstract

**Introduction:**

Pregnane X Receptor (PXR, NR1I2) is a ligand-dependent transcription factor belonging to the nuclear receptor superfamily, that can be activated by a wide variety of endogenous and exogenous ligands. It is a major actor of the endo- and xeno-biotic detoxification process. It also regulates biological processes such as lipid metabolism in large number of tissues. Pxr was shown to be expressed in human, mouse, rat and pig testis, however its roles in the regulation of testicular functions have been little explored so far.

**Methods:**

To determine the potential involvement of PXR in the regulation of steroidogenesis, experiments were performed on a wild type (MLTC-1WT) and a Pxr knock-down (MLTC-1PxrKD) mouse Leydig cell line (MLTC-1 cells), treated with a PXR agonist (SR-12813) in acute and chronic conditions.

**Results:**

Our analyses confirmed the presence of Pxr transcripts in the mouse testis, particularly in Leydig cells. In addition, A lower testosterone concentration was measured in MLTC-1PxrKD cells compared to wild type cells. Moreover, both acute and chronic stimulation of MLTC-1WT cells with SR-12813 led to a decrease in testosterone concentration, associated with a lower expression of some steroidogenic genes. This negative impact of SR-12813 on Leydig cell steroidogenesis was counteracted by Pxr knock down.

**Discussion:**

Overall, these results support the involvement of PXR in the regulation of testosterone homeostasis in mouse Leydig cells and open new avenues of research into the involvement of this receptor in the deleterious effects of certain endocrine disruptors on the steroidogenic activity of Leydig cells.

## 1 Introduction

The testis has two main functions: the production of spermatozoa and the synthesis of steroid hormones. The latter are responsible for the masculinization of the genital tract during fetal development, the acquisition of secondary sexual characteristics at puberty, and the initiation and maintenance of spermatogenesis during adulthood (1, 2). Testicular hormone production through steroidogenesis is a multi-step enzymatic process ensured in part by the Leydig cells. Their homeostasis is controlled by the hypothalamus-pituitary axis and, consequently, the main regulator of testosterone synthesis is Luteinizing Hormone (LH), which is secreted by pituitary gonadotrope cells in response to Gonadotropin-Releasing Hormone (GnRH). Following the stimulation of its receptor on the membrane of Leydig cells, the LH signaling leads to the phosphorylation and activation of the transcription factor cAMP-Response Element Binding protein (CREB) to promote the expression of steroidogenic genes. Besides CREB, the expression of genes encoding steroidogenic enzymes is also under the control of several nuclear receptors such as Steroidogenic Factor-1 (SF-1, NR5A1) (3), Liver Receptor Homolog-1 (LRH-1, Nr5a2) (4), Dosage-sensitive sex reversal, Adrenal hypoplasia critical region, on chromosome X, gene 1 (DAX-1, NR0B1) (5), Nerve Growth Factor-Induced clone B (NGFI-B/Nur77, Nr4a1) (6), Liver-X Receptors (LXRs, Nr1h2 & 3) (7), Small Heterodimer Partner (SHP, Nr0b2) (8), and Fanesoid-X Receptor α (FXRα, Nr1h4) (9). Once synthesized, testosterone exerts its action via the androgen receptor (AR), either at the testicular level or in the target tissues reached by the blood flow.

Exogenous compounds, such as endocrine disruptors, can interfere with testosterone synthesis, transport, metabolism or action, leading to abnormal development of the genital tract or infertility, depending on the exposure window (10–13). Many of them act as pro-estrogenic compounds or AR antagonists (14–16). In addition to steroid hormone receptors, some endocrine disruptors were shown to alter steroid hormone synthesis and catabolism through their binding to Peroxisome Proliferator-Activated Receptors (PPARs) (17). Next to these classical pathways, Pregnane X Receptor (PXR, NR1I2), another non-steroid hormone nuclear receptor, is a well-known target of a variety of xenobiotics, some of which acting as endocrine disruptors. It has been shown in human liver cell lines *in vitro*, that PXR is involved in activity mediated by bisphenol A (BPA) or brominated flame retardants (18). In addition, by controlling the expression of the enzyme Cytochrome P450 3A4 (CYP3A4), which plays an important role in liver steroid catabolism (19), PXR could be involved in endocrine deregulation.

PXR is a ligand-dependent transcription factor belonging to the nuclear receptor superfamily (20). It acts as a heterodimer with his partner Retinoid X Receptor (RXR) to transactivate the expression of its target genes (20). PXR was first identified as a xenobiotic sensor that regulates the expression of genes encoding drug-metabolizing enzymes and transporters to allow the detoxication and elimination of xenobiotics and endotoxins (21). It has since been involved in additional biological processes such as inflammation, glucose and lipid metabolisms as well as cellular processes among which cell proliferation, motility and apoptosis (22–29). *Pxr* is expressed in human, mouse, rat and pig testis (30–33). Consistent with its role in detoxication process, it has been shown that exposure to PXR ligands was associated with an upregulation of mRNA and protein expression of some ATP-Binding Cassette (ABC) membrane–associated drug efflux transporters expressed at the blood-testis barrier in testicular somatic Sertoli cells (30). By this way, PXR activation could limit testicular xenobiotic concentrations. In primary porcine Leydig cells, PXR transactivation led to an increase of synthesis of 16-androstenes, which are steroids stored in fat tissue (33). Thus, PXR appears to have a role in testicular functions, however it is not completely elucidated.

In the present study, we aimed to describe more precisely the potential functions of PXR in mammalian testis. We determined that *Pxr* is expressed in different testicular cell types, in particular Leydig cells, where its activation leads to a decrease of testosterone biosynthesis. These results open new research perspectives on the involvement of PXR in the deleterious effects of some endocrine disruptors on the steroidogenic activity of Leydig cells.

## 2 Materials and methods

### 2.1 Analysis of PXR expression using magnetic cell sorting in Wt male mice

Testis cell suspensions from adult Wt males were used for spermatogonia using Thy1 (Miltenyi Biotec) antibodies conjugated to MACS microbeads (Miltenyi Biotec). Briefly, testes from individual 3-month-old mice were used for adult experiment. Then, albuginea was removed, and seminiferous tubes were unwound into a dish containing 1.5 mL of M2 medium. Collagenase (20 mg mL^−1^, 50 µL) was added and incubated for 20 min at 37°C with agitation until tubes were dissociated. After removing most supernatant, 250 µL of 0.5% trypsin + 1 µL of DNAse (100 mg mL^−1^) was added to the remaining 750 µL medium and incubated for 5 min at 37°C with agitation to ensure cell dissociation. Next, 500 µL SVF was added and centrifuged for 10 min at 300 g at 4°C. The supernatant was removed, and cells were resuspended in 1 mL of sorting buffer and centrifugated for 10 min at 300 g at 4°C. Supernatant was removed, and cells were resuspended in 80 µL sorting buffer with 20 µL of the desired biotinylated antibody. The resultant solution was incubated for 15 min in a rotating cold chamber. 1 mL of sorting buffer was added to dilute antibody and then centrifuged for 10 min at 300 g at 4°C.

The supernatant was removed, and cells were recovered in 80 µL of sorting buffer. 20 µL of anti-biotin magnetic beads were added and then incubated for 15 min in a cold room on rotation. Then, 1 mL of sorting buffer was added to dilute beads and centrifuged for 10 min at 300 g at 4°C. The supernatant was then removed, and cells were recovered in 1 mL sorting buffer. After centrifugation for 10 min at 300 g at 4°C, the supernatant was removed, and cells were resuspended in 500 µL sorting buffer. The 500-µL suspension was applied to an equilibrated MACS MS column. The unretained fraction, corresponding to the unsorted cells, was collected. Thus, the eluates contain all other cell types of the testis (interstitial and tubular fractions). Columns were washed twice, and the column was removed from the magnetic rack and placed on an empty 1.5 mL tube. Then, 1 mL of sorting buffer was added, and the liquid was pressed with a plunger to recover the sorted fraction. The eluates of sorted cells, as well as the previously collected unsorted cells, were centrifugated for 10 min at 300 g at 4°C. The supernatant was removed, and cells were frozen for mRNA analysis.

### 2.2 Generation of Crispr/CAS9 *Pxr* knock-down MLTC-1 cells

*Pxr* knock-down MLTC-1 cells (MLTC-1^PxrKD^) were obtained using the Crispr/CAS9 technology and the following guides: guide 1: TTCAATGTCATGACGTGTGA and guide 2: ATCTTCCTCCTCTACGTTGA. The guides were introduced on PX458:pSpCas9(BB)-2A-GFP (PX458) (Addgene Plasmid #48138) (Addgene, Watertown, MA, UDA) and PX459: pSpCas9(BB)-2A-Puro (PX459) V2.0 (Addgene Plasmid #62988). pSpCas9(BB)-2A-Puro (PX459) was a gift from Feng Zhang (Addgene plasmid #48139). Guides were cloned on vectors at the BbsI restriction site.

MLTC-1 cells were seeded in 6-well plates and transfected with 1 µg of plasmid PX458 and 2 µg of plasmid PX459 using Jet PEI (Ozyme). The next day, MLTC-1 GFP positive cells were sorted by FACS (BD FACS Melody™ Cell Sorter from BD Biosciences) and seeded as single cell in 96-well plates in MLTC-1 conditioned medium (RPMI and 10% SVF). Clones were then validated by sequencing, genotyping and RT-qPCR using the primers reported in **Supplementary Figure S1** and **Supplementary Table S1**.

### 2.3 MLTC-1 cell culture

MLTC-1^WT^ and MLTC-1^PxrKD^ cells were cultured in RPMI medium (Fisher Scientific, Illkirch, France) supplemented with Penicillin/Streptomycin (Eurobio Scientific, Les Ulis, France), glutamin (Eurobio Scientific, Les Ulis, France) and 10% fetal bovine serum (Millipore Sigma), at 37°C in an atmosphere containing 5% CO_2_. Cells were seeded at 80 x 10^3^ cells per well in 12-well plates. The next day, the culture medium was replaced with a serum-free medium, and the cells incubated overnight before treatment with vehicle (DMSO) or the PXR agonist SR-12813 (Clinisciences, Nanterre, France) at varying concentrations and for varying times. Alternatively, cells were treated with vehicle (NaCl 0,09%) or hCG (0,125 IU) for 4h. For the experiments to analyze the AKT pathway, the cells were pre-treated for 1 hour with 10μM of the inhibitor of the PI3K, namely LY294002; and then with either hCG or SR.

### 2.4 Plasmid transfection and reporter assays

For transient transfections, 1 × 10^5^ of MLTC-1^WT^ or MLTC-1^PxrKD^ cells per well were plated in 12-well plates. On the following day, transient transfections were performed using the jetPEI^®^ transfection system (Polyplus, Illkrich, France). The *Akr1b7* (*Aldo-keto reductase 1B7*)-luciferase reporter gene either control (Akr1b7-luc) or mutated for the identified PXR-response elements (M1, M2, M3: Akr1b7-M3-luc) as using a previously described method (34, 35). Cells were co-transfected 200g of Akr1b7-luc and 800ng of empty pCMV vector. culture medium was replaced with a serum-free medium, and the cells incubated overnight before treatment with vehicle (DMSO) or the PXR agonist SR-12813 for 24 hours, in serum-free medium. The following day, the cells were lysed and luciferase activity was measured using a previously described method (36).

### 2.5 Testosterone measurement

Testosterone (MLTC-1 intra-cellular and cell culture medium) concentrations were determined by ELISA as previously performed (37) and according to the manufacturer’s instructions. In the experiments where the impacts of SR-1218 or hCG are compared between MLTC-1^WT^ and MLTC-1^PXRKD^ cells, to get a better idea of the effects of the molecules the relative concentrations have been defined in relation to a condition (in this case the vehicle). This makes it easier to compare the modulation of production induced versus untreated cell in each genotype.

### 2.6 Real-time RT-qPCR

Total RNA from MLTC-1 cells was extracted using RNAzol. cDNA was synthesized from 1 µg of RNA using the MMLV and random hexamer primers (Promega, Charbonnières-les-Bains, France). Real-time PCR reactions were run on a LightCycler Instrument Real-Time PCR System (Roche) using SYBR green dye (Master mix Plus for SYBR Assay, Takara Bio). Each PCR reaction consisted of 5 μL of SYBR green dye, 4.3 μL of water, 2 μL of cDNA sample and 0.1 μL (10 pmol) of gene-specific primers listed in **Supplementary Table S1**. Relative mRNA levels were determined with the ΔΔCt method using *Actb* as the housekeeping gene.

### 2.7 Western blotting

Proteins were extracted from cells using RIPA lysis buffer completed with protease inhibitors (Roche Diagnostics, Meylan, France). After a sonication step in chilled water bath (high intensity; two times of 30 s ON and 30 s OFF between each sonication; Bioruptor, Diagenode). The proteins were then transferred to denaturing buffer (4x Laemmli Sample Buffer, Biorad Laboratories, USA) for separation via SDS-PAGE and then transferred to nitrocellulose membrane. Subsequently, 20 μg of protein per well was used for analysis. Electrophoresis was performed in polyacrylamide gels at 120 V in denaturating buffer containing 25 mm Tris Base and 190 mm glycine, 0.1%. The antibodies used were STAR (Cell Signaling #8449) and P-CREB (Cell Signaling, #9198). For some analyses, the quantifications of the protein accumulation were performed either against housekeeping gene (Histone H3, Santa Cruz, sc8654). The band sizes and size ladder were included on western blot experiments.

### 2.8 Statistical analyses, statistical methods

Quantitative values were presented as mean± SEM. Differences between groups were determined via T-test or two-way ANOVA. When overall tests were significant (*p* < 0.05), Holm-Sidak’s *post hoc* testing was conducted for adjustment, for two-way ANOVA. Statistical analyses were conducted using Sigmastat 3.2 software as well as Prism 8.0.1. Significant differences between groups (^*^ *p* < 0.05, ^**^ *p* < 0.01, ^***^ *p* < 0.001) were defined.

## 3 Results

### 3.1 *Pxr* is expressed in the mouse testicular Leydig cells

**Figure 1:**
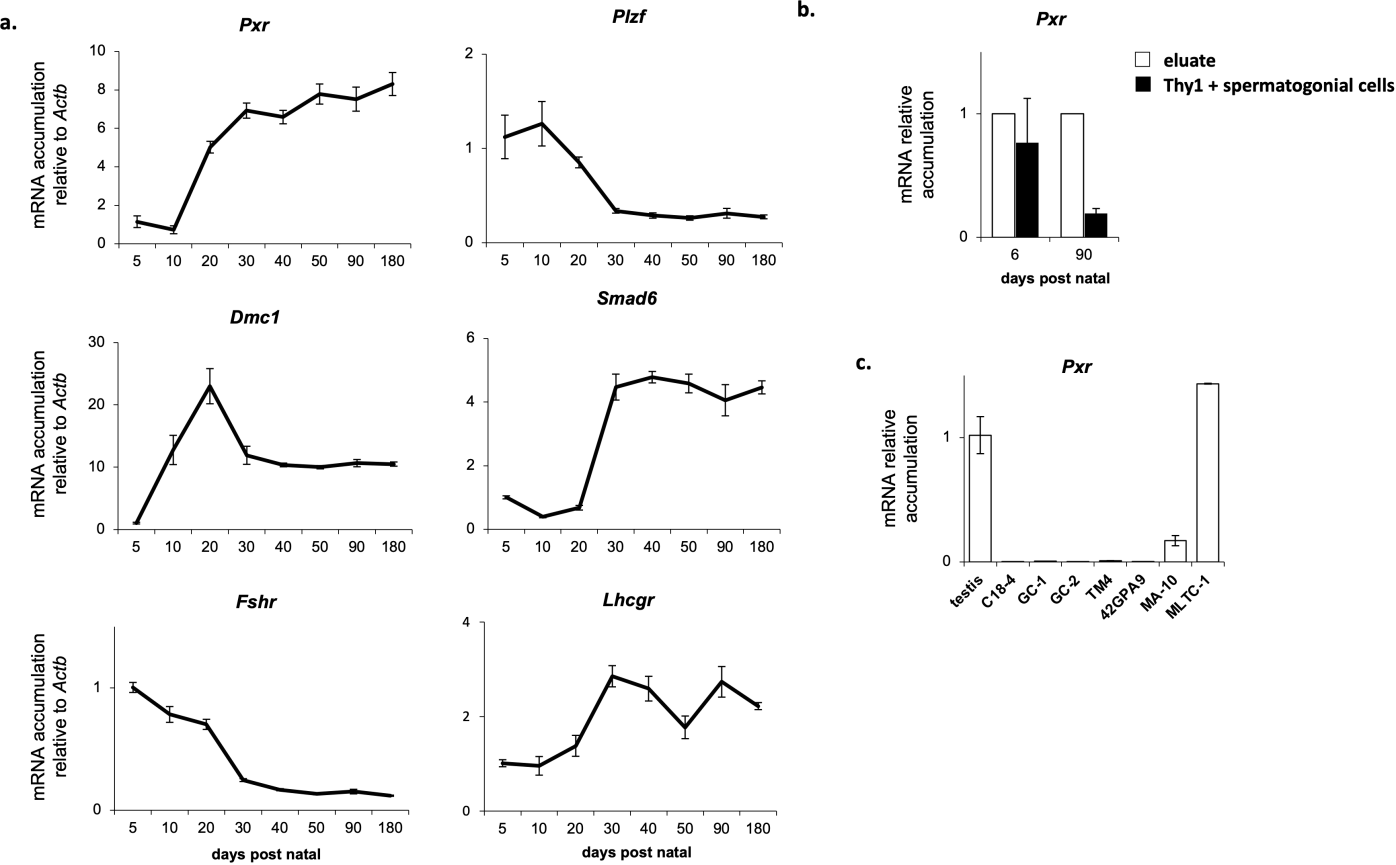
Pxr is expressed in the Leydig cells of the mouse testis. **(A)** Ontogenic mRNA accumulation of Pxr, Plzf, Dmc1, Smad6, Fshr and Lhcgr in the mouse testis. **(B)** Pxr mRNA accumulation in Thy1+ spermatogonial cells and eluate from testis of neonate and adult mice. **(C)** Pxr mRNA accumulation in several immortalized testicular cell lines. (n = 5 per group). Data are means ± SEM.

We first evaluate *Pxr* expression profile in the mouse testis. RT-qPCR analyses revealed that *Pxr* mRNA were detected in the mouse testis, from day 5 to day 180 after the birth (**Figure 1A**). *Pxr* expression in the mouse testis was low during the first 10 days of life, before increasing at 20 days of age and remaining stable in adulthood. We then compared this expression profile of *Pxr* mRNA accumulation to that of specific markers of Leydig cells (*Lhcgr*), Sertoli cells (*Fshr*), spermatogonia (*Plzf*), spermatocytes (*Dmc1*) and spermatids (*Smad6*) (**Figure 1A**). The mRNA accumulation of *Pxr* was similar to that of *Lhcgr* and *Smad6*. This as consistent with previous data where we have shown that *Pxr* is expressed in both the interstitial and tubular compartment of the mouse testis, with a higher expression level in tubular cells To determine in which cell type(s) *Pxr* is expressed in tubular compartment, its expression was further studied using THY1-positive (THY1+) sorted spermatogonia and compared with that in the eluate. In both neonatal (6 days post-natal) and adult (3 months of age) mouse testes, *Pxr* mRNA was enriched in eluate compared to THY1+ spermatogonial cells (**Figure 1B**). To further identify the testicular cell type(s) in which *Pxr* is expressed, we compared its mRNA accumulation levels between a whole testis sample and immortalized cell lines of Leydig (MA-10 and MLTC-1), Sertoli (TM4 and 42GPA9), and germ cells (C18-4, GC-1 spg and CG-2 spd(ts)). While *Pxr* mRNA are undetectable in Sertoli and germ cell lines, they are detected in the MA-10 and MLTC-1 cell lines (**Figure 1C**). Together, these results suggest that *Pxr* is predominantly expressed in Leydig.

### 3.2 *Pxr* deficient expression is associated with a deregulation of testosterone homeostasis *in vitro*

**Figure 2:**
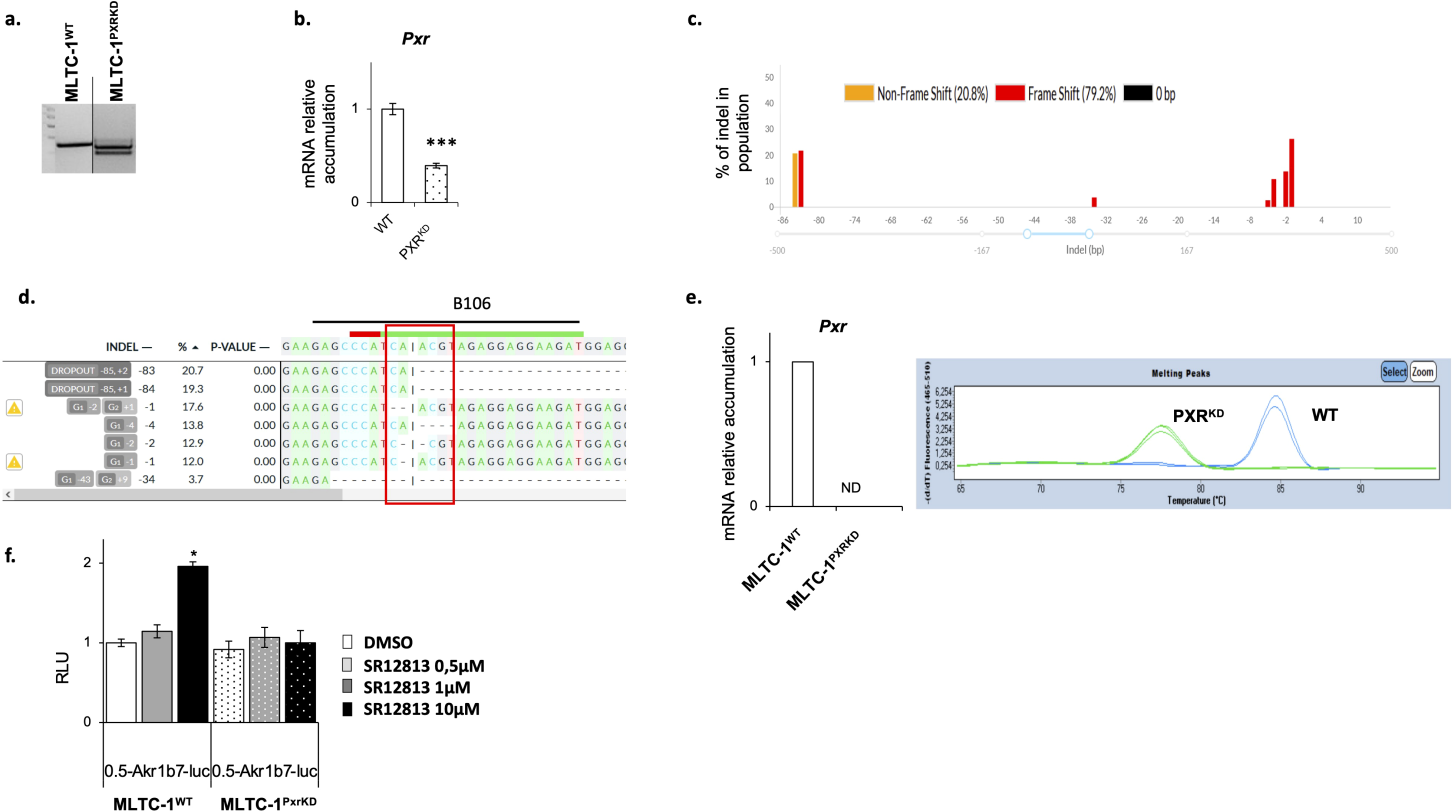
Validation of Pxr gene editing in MLTC-1 cells. **(A)** Representative image of agarose gel showing genotyping PCR. **(B)** RT-qPCR analyses of Pxr mRNA levels in wild type (WT) and PXR knock-down (PXR^KD^) MLTC-1 cells using primers allowing amplification. **(C)** Quantification of the edits present in MLTC-1^PxrKD^ cells sequencing data by DECODR software. **(D)** Sequence of the PXR genomic region at the site where CAS9 cuts (site of RNA guide, where CAS9 cuts). **(E)** RT-qPCR analyses of Pxr and representative image of melting peaks when primers (B106) at RNA guide induced sequence mutations [see panel **(D)**]. **(F)** Analysis of luciferase activity normalized to protein quantity in WT and PXR^KD^ transfected with Akr1-B7-luciferase reporter plasmid and treated 24 hours with vehicle, SR1218 at 1μM or 10μM. n=15 per group. Vehicle groups were arbitrarily set at 1. Data are means ± SEM; statistical analysis: comparisons of two groups were made using Student’s T-test and comparisons of multiple groups were done using ANOVA2: *** p<0.001.

To determine the role of PXR in Leydig cell functions, a *Pxr* mutant line was generated using the Crispr/CAS9 technology in MLTC-1 cells (Mouse Tumoral Leydig Cell Line-1 (38). To generate the mutant for *Pxr*, RNA guides were positioned to remove exon-2 (**Supplementary Figure S1**). After transfection of plasmids containing the RNA guides, the clones were selected after PCR amplification, which revealed the presence of two bands in the clone obtained after CRISPR gene editing: a band with an equivalent molecular weight to WT band, and a smaller band (**Figure 2A**). *Pxr* gene editing was validated by genotyping and Sanger sequencing (**Supplementary Figure S2**). The presence of several alleles of *Pxr* gene was confirmed with sequencing in this clone (**Supplementary Figure S2**). A deletion in the PXR genomic region was generated but as shown in **Figure 2A**, but it appeared to be a heterozygote cell line. A qPCR analysis was performed using a primer pair common to both the edited and unedited *Pxr* gene (B94/B95; see **Supplementary Figure S1**, **Supplementary Table S1**). The obtained data revealed a decrease in *Pxr* mRNA accumulation in MLTC-1^PxrKD^ cells compared with MLTC-1^WT^ cells (**Figure 2B**). These data were consistent with the fact that a heterozygous clone for PXR have been generated. However, when we carried out a more detailed analysis of the mutant clone using the Decoder software (https://decodr.org/), the analysis showed that even if a band of the same size as in the MLTC-1^WT^ was found, 100% of the DNA fragments sequenced were mutant alleles, with nucleotide deletion leading to a frameshift in 80% of these alleles (**Figure 2C**), particularly in the region targeted by the guide RNA where CAS9 was cut (**Figure 2D**). We have generated a primer (B106) corresponding this region and qPCR analyses revealed, no amplification of *Pxr* was observed when using this primer in MLTC-1^PxrKD^ cells. This was confirmed by the quantification (**Figure 2E**, left panel) and images of the qPCR analysis for which no amplicon was detected, as in the melting curves, the peak for MLTC-1^PxrKD^ corresponds to primer dimers. (**Figure 2E**, right panel). Combined, all these data validated the efficacy of gene editing. We then wanted to ensure that MLTC-1 possessed functional PXR and co-activators leading to transcriptional activation in the context of ligand treatment as well as the efficiency of genome editing. Analysis demonstrated that some of classical PXR target genes were not expressed in MLTC-1 cells such as Ugt1a1, Cyp2b10, Cyp3a25, Ugt2b34 and Sult1e (data not shown). Thus, to ensure that PXR was functional in the MLTC-1 cell line, and that this activity was lost in the generated MLTC-1^PxrKD^ cells, we tested its ability to transactivate a luciferase reporter gene driven by the *Akr1b7* gene promoter, for which effective PXR response elements (PXR-RE) have been validated. The A*kr1b7* promoter construct including −510 bp to +41 bp (0.5 *akr1b7*-luc) was used (34, 35). Data showed that the luciferase activity was induced by cell treatment with SR-1218 (**Figure 2F**). In contrast, no effect of the SR-1218 was observed when cells were transfected in MLTC-1^PxrKD^ cells transfected with the wild-type 0.5 *Akr1b7*-luc construct. Combined these data highlighted the fact that PXR signaling is efficient in MLTC-1 cells and that our gene editing led to a loss of function of PXR in the MLTC-1 cell line (**Figure 2F**).

**Figure 3:**
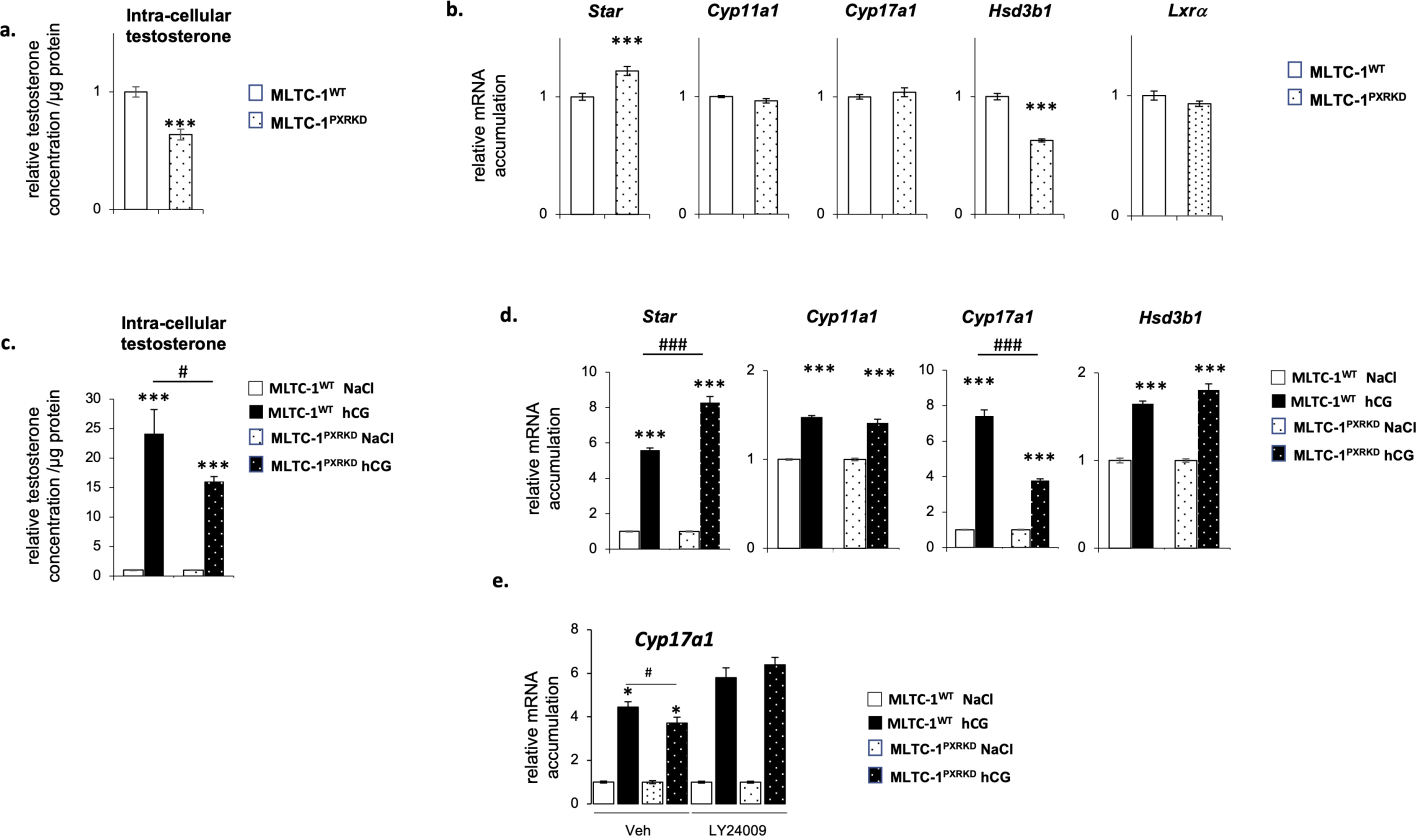
Pxr deficiency is associated with a deregulation of testosterone homeostasis in vitro. **(A)** Testosterone concentration in cells corrected to protein concentration in MLTC-1^WT^ and MLTC-1^PxrKD^ cells 24h after serum starvation. **(B)** mRNA accumulation of Star, Cyp11a1, Cyp17a1, Hsd3b1 and Lxrα normalized to b-actin in MLTC-1^WT^ and MLTC-1^PxrKD^ cells 24h after serum starvation. **(C)** Testosterone concentration in cells corrected to protein concentration in MLTC-1^WT^ and MLTC-1^PxrKD^ cells treated for 4h with vehicle or 0,125 IU hCG. **(D)** mRNA accumulation of Star, Cyp11a1, Cyp17a1 and Hsd3b1 in MLTC-1^WT^ and MLTC-1^PxrKD^ cells treated for 4h with vehicle or 0,125 IU hCG. **(E)** mRNA accumulation of Cyp17a1 in MLTC-1^WT^ and MLTC-1^PxrKD^ cells pre-treated for 1h with LY24009 at 10μM and then for 4h with vehicle or 0,125 IU hCG. n =3 triplicates per group; data means ± SEM; statistical analysis: comparisons of two groups were made using Student’s T-test and comparisons of multiple groups were done using ANOVA2: * p<0.05; *** p<0.001; # p<0.05; ### p<0.001.

To analyze the role of PXR on testicular steroidogenesis, testosterone concentration was measured in both MLTC-1^WT^ and MLTC-1^PxrKD^ cells, 24h after serum starvation. A lower intra-cellular testosterone concentration was measured in MLTC-1^PxrKD^ cells compared to MLTC-1^WT^ cells (**Figure 3A**). To decipher the origin of the altered testosterone production, we then determined the expression level of genes encoding steroidogenic enzymes by RT-qPCR in MLTC-1^WT^ and MLTC-1^PxrKD^ cell lines. Among all the genes tested, a decrease of mRNA accumulation of *Hsd3b1* was noted in MLTC-1^PxrKD^ cells compared to MLTC-1^WT^ cells (**Figure 3B**). As in basal condition, only *Hsd3b1* was affected in MLTC-1^PxrKD^ cells compared to MLTC-1^WT^ cells, we wondered whether crosstalk with the Liver-X-Receptor alpha (LXRα) might exist. Indeed, it has been shown that in Lxrα knock-out mouse testis, in which testosterone is affected, only *Hsd3b1* expression was decreased due to the absence of *Lxrα* (7). We therefore tested the level of *Lxrα* in MLTC-1^PxrKD^ cells, but no difference was observed compared with MLTC-1^WT^ cells (**Figure 3B**). This hypothesis has therefore not been validated and it remains to be determined how PXR regulates *Hsd3b1* expression.

The present data also demonstrated that MLTC-1^PxrKD^ cells were less sensitive to LH/CG signaling than MLTC-1^WT^ cells, as they produced a smaller amount of testosterone in response to hCG stimulation (**Figure 3C**). At the molecular level, this was associated with a lower induction of *Cyp17a1* expression in response to hCG (**Figure 3D**). This effect appeared to be limited to *Cyp17a1* as no impact was observed on *Star* and *Cyp11a1*. Interestingly, PI3K/Akt pathways have been described as having a specific impact on the sensitivity of the Cyp17a1 gene to hCG (39). We therefore decided to test this hypothesis. MLTC-1^WT^ and MLTC-1^PxrKD^ cells were pre-treated with an inhibitor of the PI3K, namely LY24009, and then treated for 4 hours with hCG (**Figure 3E**). As expected, a lower induction of the expression of *Cyp17a1* in response to hCG was observed in MLTC-1^PxrKD^ cells compared with MLTC-1^WT^ cells, but this difference was not observed in cells pretreated with LY24009. Indeed, in this condition, the sensitivity of MLTC-1^PxrKD^ cells was identical to that of MLTC-1^WT^ cells. This confirms that PXR could act via the PI3K/AKT pathway, although the exact molecular mechanisms remain to be defined in Leydig cells. However, this link between PXR and the PI3K/AKT signaling pathway is entirely consistent with the fact that PXR was associated with multiple crucial signaling pathways, especially the PI3K/AKT pathway (40). Together, these results suggest a role of PXR in the regulation of testosterone homeostasis in Leydig cells.

### 3.3 PXR agonist, SR-12813, leads to a decrease of testosterone concentration in Leydig cells

To further determine the role of PXR in mouse Leydig cells, we decided to modulate its activity using a selective synthetic agonist, namely SR-12813 (41) in order to decipher its impact on testosterone homeostasis.

**Figure 4:**
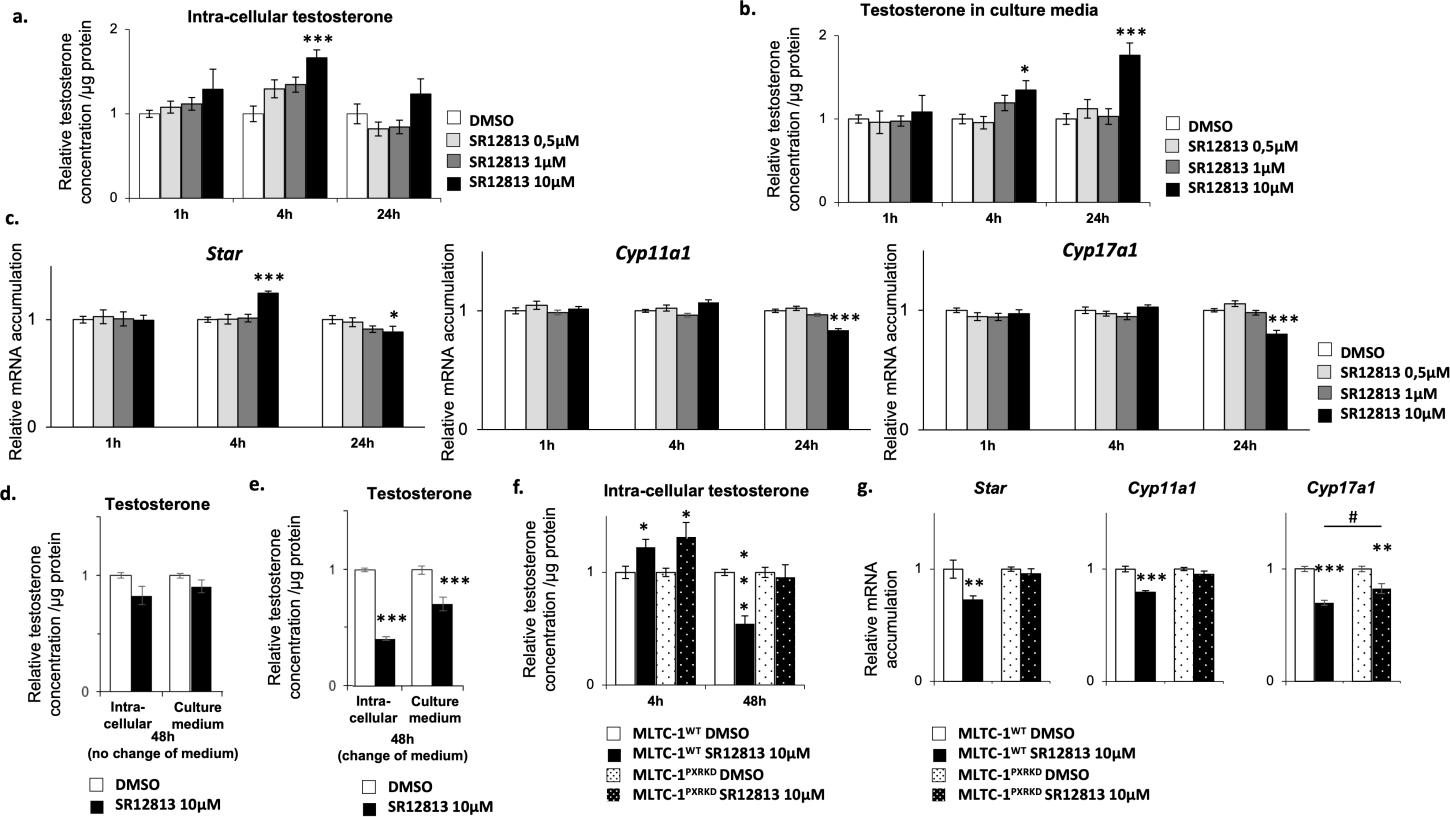
SR-12813 leads to a decrease of testosterone concentration in Leydig cells in a PXR dependent manner. MLTC-1^WT^ cells were treated for 1, 4 and 24h with vehicle or 0,5, 1 and 10 µM SR-12813 to determine testosterone concentration in **(A)** cells (corrected to protein concentration) and **(B)** culture media, and **(C)** expression of Star, Cyp11a1, Cyp17a1 and Hsd3b1 by RT-qPCR. MLTC-1^WT^ cells were treated for 48h with vehicle or 10 µm SR-12813 to determine testosterone concentration in cells (corrected to protein concentration) and culture media **(D)** without and **(E)** with a medium change after 24h of treatment. **(F)** Intra-cellular testosterone concentration corrected to protein concentration from MLTC-1^WT^ and MLTC-1^PxrKD^ cells treated with vehicle or 10 µM SR-12813 for 4 or 48h (medium being changed after 24h). **(G)** mRNA accumulation of Star, Cyp11a1 and Cyp17a1 normalized to b-Actin from MLTC-1^WT^ and MLTC-1^PxrKD^ treated with vehicle or 10 µM SR-12813 for 24h. n =3 triplicates per group; data means ± SEM; statistical analysis: comparisons of two groups were made using Student’s T-test and comparisons of multiple groups were done using ANOVA2: *** p<0.001, # p<0.05.

The first approach used was to define the doses at which SR-1218 was efficient. For that purpose, MLTC-1^WT^ cells were treated with graded concentrations of SR-12813 for 1h, 4h, and 24h. Experiments showed that the addition of SR-12813 at the concentration of 1 and 10 µM led to a transient increase of intra-cellular testosterone levels after 4 hours (**Figure 4A**). A significant increase of testosterone concentration in culture media was measured from 4 to 24 h of treatment when SR-12813 was used at 10µM, which might reflect the accumulation of testosterone from early increased production (**Figure 4B**). Consistently with these results, the addition of SR-12813 at 10 µM led to an increase of mRNA accumulation levels of *Star* and *Cyp11a1* after 4h of treatment (**Figure 4C**).

Interestingly, mRNA accumulation levels of *Star*, *Cyp11a1* and *Cyp17a1* diminished after 24h of treatment when SR-12813 was used at 10 µM (**Figure 4C**). We wondered whether this reduction in the expression of steroid synthesis genes could lead to a long-term decrease in testosterone production. Interestingly, a downward trend in testosterone concentration was noted after 48 hours of treatment with 10 μM SR-12813 (**Figure 4D**). The different impact of SR-12813 in culture media at 24h and 48h (**Figures 4B, D**) suggested that treated cells might have reduced their testosterone synthesis between these two timings of analysis. Indeed, at these timings, testosterone concentration in the medium reflects the overall level of its production and accumulation, as testosterone is not metabolized by Leydig cells. Thus, to determine the rate of testosterone production during the last 24h of treatment (between 24h to 48h), we removed the culture media 24h after the beginning of treatment and replaced it with fresh medium for an additional 24h. By this way, we were able to detect a significant decrease of testosterone concentration in cells and culture media (**Figure 4E**), supporting the idea that testosterone production was decreased in these last 24h. These results were consistent with the decrease of *Star*, *Cyp11a1* and *Cyp17a1* mRNA accumulation levels observed after 24h of treatment (**Figure 4C**). Combined all these data suggested that the SR-1218 was the most efficient at 10µM. Thus, we used this particular dose of 10µM of SR-12813 and protocol (change of medium), to determine if it acts via PXR, MLTC-1^WT^ and MLTC-1^PxrKD^ cells were treated for 4h and 48h with SR-1218. As expected, the addition of SR-12813 promoted testosterone production after 4h in MLTC-1^WT^ cells, and surprisingly this effect was not mediated by PXR as it was also observed in MLTC-1^PxrKD^ cells (**Figure 4F**). However, intra-cellular testosterone levels reduction observed in MLTC-1^WT^ cells after 48h of treatment was completely abolished in MLTC-1^PxrKD^ cells. These data support the role of PXR as an inhibitor of steroid synthesis. Consistently, the decrease of mRNA accumulation levels of genes encoding steroidogenic enzymes observed in MLTC-1^WT^ cells after 24h of treatment was completely (*Star* and *Cyp11a1*) or partially (*Cyp17a1*) counteracted by *Pxr* knock down (**Figure 4G**).

One could hypothesize that some effect of SR-1218 could be dependent of CREB as it has been defined as a major regulator of steroidogenic genes. Moreover, it has been demonstrated in liver cells that PXR could interact directly with CREB DBD to inhibit its transcriptional activity, as it can with other transcription factors. The crosstalk between PXR and CREB has been demonstrated in the liver, where in PXR-knock-out there is a decrease of CREB pathway leading to a decrease of the mRNA accumulation of G6Pase (27). On MTLC-1 cells, an opposite result was observed compared to liver cells with a huge increase (more than 3-fold) of G6Pase in MLTC-1^PxrKD^ cells (**Supplementary Figure S3A**). However, we tested this and no impact of PXR was noticed on the accumulation of P-CREB is altered in PXR^KD^ vs WT in the context of SR-1218 or hCG inductions (**Supplementary Figures S3B, C**). Thus, the hypothesis of a crosstalk between PXR and CREB seemed to be unlikely the cause of the decrease mRNA accumulation of *Star*, *Cyp11a1* and *Cyp17a1*, thus further investigations are needed.

### 3.4 Chronical activation of PXR leads to a decrease of testosterone concentration in Leydig cells

**Figure 5:**
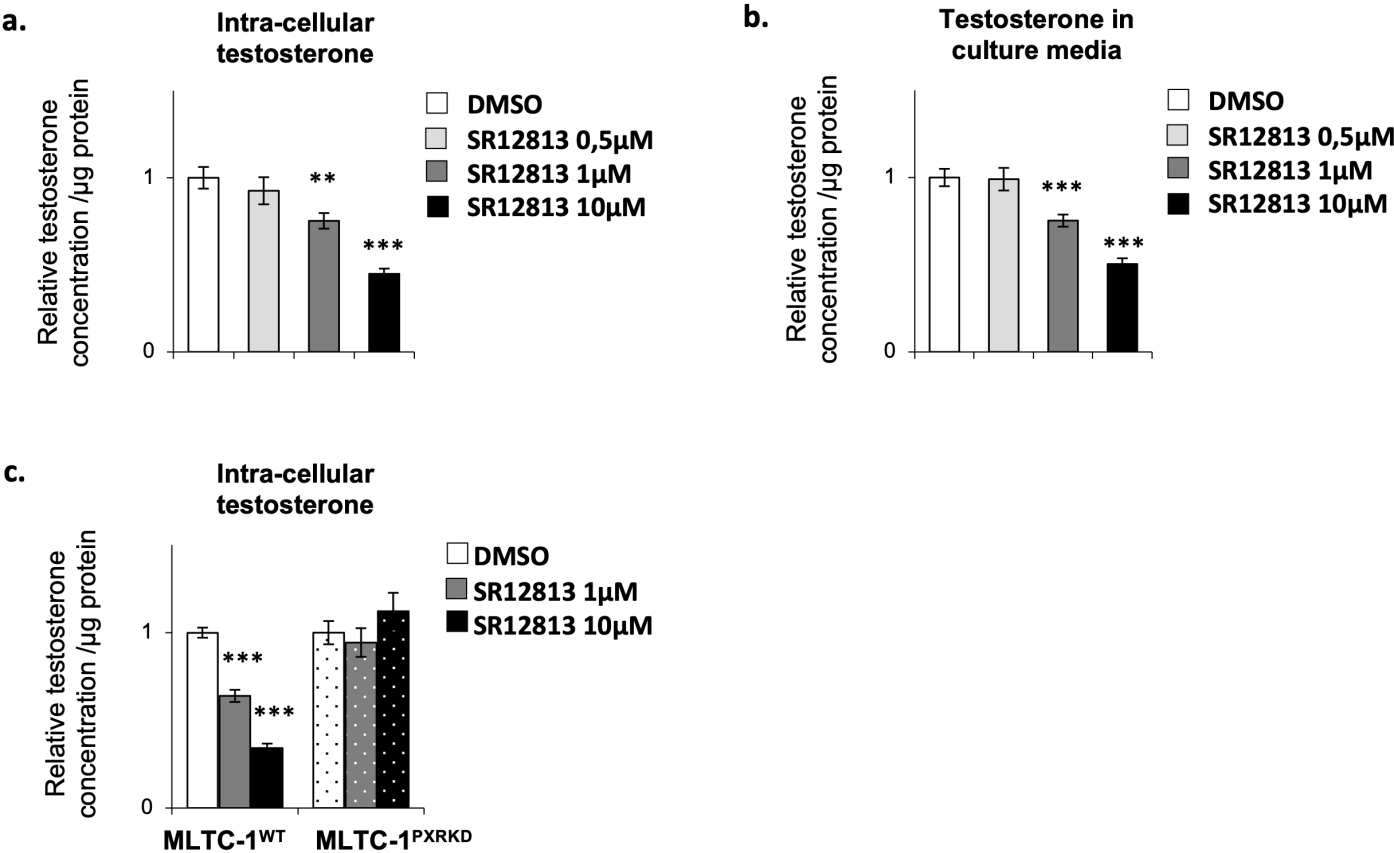
Chronical activation of PXR leads to a decrease of testosterone concentration in Leydig cells. **(A)** Intra-cellular testosterone concentration corrected to protein concentration from MLTC-1^WT^ cells treated every 24h for 3 days with vehicle or 0,5, 1 and 10 µM SR-12813. **(B)** Culture media testosterone concentration corrected to protein concentration from MLTC-1 cells treated every 24h for 3 days with vehicle or 0,5, 1 and 10 µM SR-12813. **(C)** Intra-cellular testosterone concentration corrected to protein concentration from MLTC-1^WT^ and MLTC-1^PxrKD^ cells treated every 24h for 3 days with vehicle or 0,5, 1 and 10 µM SR-12813. n =3 triplicates per group; data are means ± SEM; statistical analysis (Student’s T-test): ** p<0.01; *** p<0.001.

As the sensitivity of Leydig cells to stimulus can be different whether the stress is acute or chronic (42), we decided to evaluate the impact of a chronic stimulation of Leydig cells with SR-12813 on their steroidogenic function. MLTC-1^WT^ cells were thus treated chronically, by adding 0,5, 1 and 10 µM of SR-12813 every 24h for 3 days, and testosterone concentration were measured in intra-cellular extracts and culture media. A dose-dependent decrease of testosterone levels in both cells and culture media was observed in MLTC-1^WT^ cells (**Figures 5A, B**). This effect was only significant when SR-12813 was used at 1 and 10 µM. Interestingly, no effect was observed in MLTC-1^PxrKD^ cells treated chronically at 1 and 10 µM of SR-12813 (**Figure 5C**). These data clearly support the identification of a key role for PXR in the regulation of testosterone synthesis by Leydig cells.

## 4 Discussion

PXR is a member of the nuclear receptor family involved in the control of numerous functions such as endo- and xeno-biotic metabolism or inflammation (21, 22). Although it has been described as being expressed in the testicles (31), its role in testicular functions has not been completely elucidated yet. Our data show that *Pxr* is expressed in multiple cell types of the mouse testis. On the one hand, *Pxr* is expressed in tubular cell extracts. mRNA from *Pxr* were almost undetectable in Sertoli, spermatogonial and spermatocyte cell lines. These data suggest that *Pxr* may be expressed in post-meiotic germ cells in which it could play a role in the regulation of spermatogenesis, which has not been explored so far. On the other hand, the present data highlight the expression of *Pxr* in mouse Leydig cells, what led us to analyze its role in the control of testicular endocrine function. In the present study, using Cripr/CAS9 cell editing, we show that a deregulation of Pxr expression and activity in the mouse Leydig tumor cell line MLTC-1 leads to an alteration of the expression of some steroidogenic genes and in turn of testosterone concentration. The activation of PXR by SR-12813 leads to a drop of mRNA accumulation level of *Star*, *Cyp11a1* and *Cyp17a1* that precedes a decrease of testosterone levels. In addition, the present data show that the PXR plays a role in the regulation of testosterone synthesis in interaction with the LH/C pathways, since administration of SR-11218 reduces the sensitivity of Leydig cells to stimulation by hCG. We thus propose that PXR represents a novel actor involved in the regulation of Leydig cell steroidogenesis. It is interesting to note that some steroid derivatives (pregnanes) were identified as natural endogenous ligands for PXR (20). We could thus hypothesize that PXR may participate in a negative feedback loop that allows steroids to regulate their own synthesis in physiologic conditions.

The fact that PXR could control the endocrine function of the testis opens a new path of research perspectives with potential health impacts. Indeed, over the last decades, PXR has been defined as a receptor of xenobiotics. It is activated by a wide variety of xenobiotics, including environmental endocrine disruptors [bisphenols, phthalates, per- and polyfluoroalkyl Substances (43–45)], that are known to alter hormonal homeostasis. However, the mechanisms of action by which PXR is thought to be involved in endocrine disruption are still poorly defined. Indeed, some studies suggest that PXR may take part in liver mechanisms initiated by endocrine disruptors to alter steroid homeostasis. Indeed, PXR has been involved in the activation of Cyp3A4 expression or activity mediated by BPA or brominated flame retardants in human liver cell lines. Because the CYP3A4 enzyme plays an important role in steroid catabolism, induction of its expression or activity may lead to endocrine disruption. Moreover, Zhang et al. showed that genetic and pharmacological activation of PXR inhibited androgen-dependent prostate regeneration in castrated male mice that received daily injections of testosterone propionate by inducing the liver expression of Cytochrome P450 (CYP)3As and Hydroxysteroid Sulfotransferase (SULT)2A1, which are important enzymes for the metabolic deactivation of androgens (46). Next to this, our data suggest that PXR could lead to endocrine disruption in another way. Indeed, the present data show that PXR might also mediate the negative impact of endocrine disruptors on hormonal homeostasis by directly inhibiting testicular steroidogenesis. Furthermore, here we show that a chronic activation of PXR leads to a greater decrease of testosterone concentration than an acute stimulation of PXR activity. This might be important in the context of chronic exposure to environmental pollutant throughout life.

If we define the negative impact of PXR on testosterone synthesis in mouse Leydig cells, the molecular mechanisms are not precisely defined and need further investigations. One possibility could be that PXR interferes with the transcriptional activity of CREB to lower mRNA accumulation of the genes encoding the steroidogenic enzymes *Star*, *Cyp11a1* and *Cyp17a1*. Indeed, Kodama et al. showed in a human hepatocarcinoma cell line and in mouse primary hepatocytes that PXR repressed the basal level and the cAMP-induced increase expression of *G6Pase* and *Pepck1* genes by directly binding to CREB and thus by preventing its interaction with the cAMP-Response Element (CRE) (27), leading to a decrease in G6Pase mRNA accumulation. However, in MLTC-1 cells, the lack of PXR leads to the opposite result with a considerable (more than 3-fold) increase in G6Pase (**Supplementary Figure S3A**). Furthermore, if such crosstalk between PXR and CREB exists, one would expect a similar effect on all steroidogenic genes since they have all been described as targets of the PKA-CREB pathway. However, this is not the case, since at basal level, only Hsd3b1 was affected by the absence of PXR, and upon stimulation with hCG, only the accumulation of Cyp17a1 mRNA was affected in PXR KD cells compared with WT cells. Thus, all these data combined suggest that it is very unlikely that there is an alteration in the CREB pathway under basal or hCG-induced conditions. However, we tested this and no impact of PXR was noticed on P-CREB accumulation (**Supplementary Figure S3B**). In addition, no impact of SR1218 was seen on P-CREB. The mechanism of the link between PXR and the regulation of steroidogenesis has not yet been fully established and will require additional research.

It has been demonstrated that the regulation of steroidogenesis can also depends on post-translational modification, for STAR. However, no difference was observed on phosphorylation of STAR between MLTC-1^WT^ and MLTC-1^PxrKD^ cells either in basal condition or even in the context of hCG or SR-1218 treatments. However, previous work using MLTC-1 cell line, where the authors showed that in mouse Leydig cells, cAMP-independent signaling events regulate steroidogenesis in a StAR phosphorylation-independent manner (47).

However, under the conditions of treatment with HCG, we were able to define that the inhibitory impact of the absence of PXR on the sensitivity of cells to hCG seems to involve the PI3K/AKT pathway, as suggested using the inhibitor LY204009. This is consistent with previously published data showing that the addition of LY24009 only impacted the action of hCG on CYP17 expression (39). This link between PXR and the AKT signaling pathway is entirely consistent with the fact that PXR was associated with multiple crucial signaling pathways, especially the PI3K/AKT pathway (40).

The present data also highlight some impacts of the SR-12813 in a PXR-independent manner, with a transient increase of mRNA accumulation levels of *Star* and *Cyp11a1* and of testosterone concentration in MLTC-1 cells. It is well known that SR-12813 could have effect by increasing degradation of 3-Hydroxy-3-Methylglutaryl-Coenzyme A Reductase (HMG-CoA-Red) (48) or by activating the nuclear receptor Farnesoid X Receptor (FXRα) at µM concentrations. In both cases, it is not evident to explain the increased testosterone production. Indeed, on one hand the inhibition of HMG-CoA-Red might decrease the level of cholesterol, which is the substrate for steroidogenesis (49). On the other hand, the activation of FXRα was associated with a repression of testosterone synthesis both *in vivo* and *in vitro* (9).

### 4.1 Identified limitations of the present study

Although we have clearly demonstrated the impact of the PXR signaling pathway on Leydig cells, this work is mainly based on *in vitro* experiments. This is a good start in highlighting the role of PXR in controlling endocrine function. We need to be cautious, as there may be some differences between data from cell lines, Leydig cells sorted *in vivo* or primary cell cultures. In addition, further studies are needed to decipher the impact *in vivo* on physiological processes, whether during development with the major impact of sex steroids on sexual maturation or in adulthood for the maintenance of spermatogenesis and fertility. Furthermore, at the molecular level, additional data concerning the role of PXR in Leydig cells should be collected. As PXR is a nuclear receptor and consensus sequences of PXR response elements have been identified, Chip-seq approaches combined with transcriptomic analysis on Leydig cells would be interesting to determine its target genes in this context. Several endobiotics and xenobiotics can modulate PXR activity, and our current data was only obtained with one specific molecule. Thus, we cannot extrapolate the differences that might be observed with other PXR ligands. Some previous studies have highlighted the major difference between the PXR receptor in mice and humans, with molecules presenting specific interactions. Thus, the responses observed in the mouse cell line could differ from those observed in humans. These limitations must be considered in future experiments.

## 5 Conclusion

As a conclusion, we have identified the nuclear receptor PXR as a new actor that may participate in the regulation of Leydig cell steroidogenesis. As PXR can be activated by a wide variety of xenobiotics, these results open new research perspectives on the involvement of PXR in the deleterious effects of some endocrine disruptors on the steroidogenic activity of Leydig cells.

## Data Availability

The raw data supporting the conclusions of this article will be made available by the authors, without undue reservation.

## References

[1] Wilson JD Griffin JE George FW Leshin M. The endocrine control of male phenotypic development. Aust J Biol Sci. (1983) 36:101−28. doi: 10.1071/BI9830101 6354161

[2] Singh J O’Neill C Handelsman DJ. Induction of spermatogenesis by androgens in gonadotropin-deficient (hpg) mice. Endocrinology. (1995) 136:5311−21. doi: 10.1210/endo.136.12.7588276 7588276

[3] Parker KL Schimmer BP. Steroidogenic factor 1: a key determinant of endocrine development and function. Endocr Rev. (1997) 18:361−77. doi: 10.1210/edrv.18.3.0301 9183568

[4] Kim JW Peng N Rainey WE Carr BR Attia GR. Liver receptor homolog-1 regulates the expression of steroidogenic acute regulatory protein in human granulosa cells. J Clin Endocrinol Metab. (2004) 89:3042−7. doi: 10.1210/jc.2003-031599 15181096

[5] Zazopoulos E Lalli E Stocco DM Sassone-Corsi P. DNA binding and transcriptional repression by DAX-1 blocks steroidogenesis. Nature. (1997) 390:311−5. doi: 10.1038/36899 9384387

[6] Zhang P Mellon SH. Multiple orphan nuclear receptors converge to regulate rat P450c17 gene transcription: novel mechanisms for orphan nuclear receptor action. Mol Endocrinol. (1997) 11:891−904. doi: 10.1210/mend.11.7.9940 9178749

[7] Volle DH Mouzat K Duggavathi R Siddeek B Déchelotte P Sion B. Multiple roles of the nuclear receptors for oxysterols liver X receptor to maintain male fertility. Mol Endocrinol. (2007) 21:1014−27. doi: 10.1210/me.2006-0277 17341595

[8] Volle DH Duggavathi R Magnier BC Houten SM Cummins CL Lobaccaro JMA. The small heterodimer partner is a gonadal gatekeeper of sexual maturation in male mice. Genes Dev. (2007) 21:303−15. doi: 10.1101/gad.409307 17289919 PMC1785120

[9] Baptissart M Martinot E Vega A Sédes L Rouaisnel B de Haze A. Bile acid-FXRα pathways regulate male sexual maturation in mice. Oncotarget. (2016) 7:19468−82. doi: 10.18632/oncotarget.v7i15 26848619 PMC4991395

[10] Gill WB Schumacher GF Bibbo M. Pathological semen and anatomical abnormalities of the genital tract in human male subjects exposed to diethylstilbestrol. utero J Urol. (1977) 117:477−80. 850321 10.1016/s0022-5347(17)58502-x

[11] Swan SH Main KM Liu F Stewart SL Kruse RL Calafat AM. Decrease in anogenital distance among male infants with prenatal phthalate exposure. Environ Health Perspect. (2005) 113:1056−61. doi: 10.1289/ehp.8100 16079079 PMC1280349

[12] Wirth JJ Rossano MG Potter R Puscheck E Daly DC Paneth N. A pilot study associating urinary concentrations of phthalate metabolites and semen quality. Syst Biol Reprod Med. (2008) 54:143−54. doi: 10.1080/19396360802055921 18570050

[13] Pant N Shukla M Kumar Patel D Shukla Y Mathur N Kumar Gupta Y. Correlation of phthalate exposures with semen quality. Toxicol Appl Pharmacol. (2008) 231:112−6. doi: 10.1016/j.taap.2008.04.001 18513777

[14] Kasongo AA Leroux M Amrouche-Mekkioui I Belhadji-Domecq M Aguer C. BPA exposure in L6 myotubes increased basal glucose metabolism in an estrogen receptor-dependent manner but induced insulin resistance. Food Chem Toxicol. (2022) 170:113505. doi: 10.1016/j.fct.2022.113505 36328215

[15] Babiloni-Chust I Dos Santos RS Medina-Gali RM Perez-Serna AA Encinar JA Martinez-Pinna J. G protein-coupled estrogen receptor activation by bisphenol-A disrupts the protection from apoptosis conferred by the estrogen receptors ERα and ERβ in pancreatic beta cells. Environ Int. (2022) 164:107250. doi: 10.1016/j.envint.2022.107250 35461094

[16] Lemaire G Terouanne B Mauvais P Michel S Rahmani R. Effect of organochlorine pesticides on human androgen receptor activation. vitro Toxicol Appl Pharmacol. (2004) 196:235−46. doi: 10.1016/j.taap.2003.12.011 15081270

[17] Corton JC Lapinskas PJ. Peroxisome proliferator-activated receptors: mediators of phthalate ester-induced effects in the male reproductive tract? Toxicol Sci. (2005) 83:4−17. doi: 10.1093/toxsci/kfi011 15496498

[18] Kuzbari O Peterson CM Franklin MR Hathaway LB Johnstone EB Hammoud AO. Comparative analysis of human CYP3A4 and rat CYP3A1 induction and relevant gene expression by bisphenol A and diethylstilbestrol: implications for toxicity testing paradigms. Reprod Toxicol. (2013) 37:24−30. doi: 10.1016/j.reprotox.2013.01.005 23384967

[19] Waxman DJ Attisano C Guengerich FP Lapenson DP. Human liver microsomal steroid metabolism: identification of the major microsomal steroid hormone 6 beta-hydroxylase cytochrome P-450 enzyme. Arch Biochem Biophys. (1988) 263:424−36. doi: 10.1016/0003-9861(88)90655-8 3259858

[20] Kliewer SA Moore JT Wade L Staudinger JL Watson MA Jones SA. An orphan nuclear receptor activated by pregnanes defines a novel steroid signaling pathway. Cell. (1998) 92:73−82. doi: 10.1016/S0092-8674(00)80900-9 9489701

[21] Rosenfeld JM Vargas R Xie W Evans RM. Genetic profiling defines the xenobiotic gene network controlled by the nuclear receptor pregnane X receptor. Mol Endocrinol. (2003) 17:1268−82. doi: 10.1210/me.2002-0421 12663745

[22] Zhou C Tabb MM Nelson EL Grün F Verma S Sadatrafiei A. Mutual repression between steroid and xenobiotic receptor and NF-kappaB signaling pathways links xenobiotic metabolism and inflammation. J Clin Invest. (2006) 116:2280−9. doi: 10.1172/JCI26283 16841097 PMC1501109

[23] Shizu R Benoki S Numakura Y Kodama S Miyata M Yamazoe Y. Xenobiotic-induced hepatocyte proliferation associated with constitutive active/androstane receptor (CAR) or peroxisome proliferator-activated receptor α (PPARα) is enhanced by pregnane X receptor (PXR) activation in mice. PloS One. (2013) 8:e61802. doi: 10.1371/journal.pone.0061802 23626729 PMC3634023

[24] Ouyang N Ke S Eagleton N Xie Y Chen G Laffins B. Pregnane X receptor suppresses proliferation and tumorigenicity of colon cancer cells. Br J Cancer. (2010) 102:1753−61. doi: 10.1038/sj.bjc.6605677 20531417 PMC2883694

[25] Niu Y Wang Z Huang H Zhong S Cai W Xie Y. Activated pregnane X receptor inhibits cervical cancer cell proliferation and tumorigenicity by inducing G2/M cell-cycle arrest. Cancer Lett. (2014) 347:88−97. doi: 10.1016/j.canlet.2014.01.026 24486740

[26] Kodama S Negishi M. Pregnane X receptor PXR activates the GADD45beta gene, eliciting the p38 MAPK signal and cell migration. J Biol Chem. (2011) 286:3570−8. doi: 10.1074/jbc.M110.179812 21127053 PMC3030361

[27] Kodama S Moore R Yamamoto Y Negishi M. Human nuclear pregnane X receptor cross-talk with CREB to repress cAMP activation of the glucose-6-phosphatase gene. Biochem J. (2007) 407:373−81. doi: 10.1042/BJ20070481 17635106 PMC2275060

[28] Yan L Yang K Wang S Xie Y Zhang L Tian X. PXR-mediated expression of FABP4 promotes valproate-induced lipid accumulation in HepG2 cells. Toxicol Lett. (2021) 346:47−56. doi: 10.1016/j.toxlet.2021.04.016 33901630

[29] Li T Chen W Chiang JYL. PXR induces CYP27A1 and regulates cholesterol metabolism in the intestine. J Lipid Res. (2007) 48:373−84. doi: 10.1194/jlr.M600282-JLR200 17088262

[30] Whyte-Allman SK Hoque MT Jenabian MA Routy JP Bendayan R. Xenobiotic nuclear receptors pregnane X receptor and constitutive androstane receptor regulate antiretroviral drug efflux transporters at the blood-testis barrier. J Pharmacol Exp Ther. (2017) 363:324−35. doi: 10.1124/jpet.117.243584 28970358

[31] Martinot E Baptissart M Véga A Sèdes L Rouaisnel B Vaz F. Bile acid homeostasis controls CAR signaling pathways in mouse testis through FXRalpha. Sci Rep. (2017) 7:42182. doi: 10.1038/srep42182 28181583 PMC5299845

[32] Tully DB Bao W Goetz AK Blystone CR Ren H Schmid JE. Gene expression profiling in liver and testis of rats to characterize the toxicity of triazole fungicides. Toxicol Appl Pharmacol. (2006) 215:260−73. doi: 10.1016/j.taap.2006.02.015 16643972

[33] Gray MA Squires EJ. Effects of nuclear receptor transactivation on steroid hormone synthesis and gene expression in porcine Leydig cells. J Steroid Biochem Mol Biol. (2013) 133:93−100. doi: 10.1016/j.jsbmb.2012.09.014 23000191

[34] Volle DH Repa JJ Mazur A Cummins CL Val P Henry-Berger J. Regulation of the aldo-keto reductase gene akr1b7 by the nuclear oxysterol receptor LXRα (Liver X receptor-α) in the mouse intestine: putative role of LXRs in lipid detoxification processes. Mol Endocrinol. (2004) 18:888−98. doi: 10.1210/me.2003-0338 14739254

[35] Liu MJ Takahashi Y Wada T He J Gao J Tian Y. The aldo-keto reductase Akr1b7 gene is a common transcriptional target of xenobiotic receptors pregnane X receptor and constitutive androstane receptor. Mol Pharmacol. (2009) 76:604−11. doi: 10.1124/mol.109.057455 19542321 PMC2730391

[36] Thirouard L Holota H Monrose M Garcia M de Haze A Damon-Soubeyrand C. Identification of a crosstalk among TGR5, GLIS2, and TP53 signaling pathways in the control of undifferentiated germ cell homeostasis and chemoresistance. Adv Sci (Weinh). (2022) 9:e2200626. doi: 10.1002/advs.202200626 35435331 PMC9189661

[37] Holota H De Haze A Martinot E Monrose M Saru JP Caira F. Identification of the role of TGR5 in the regulation of leydig cell homeostasis. Int J Mol Sci. (2022) 23:15398. doi: 10.3390/ijms232315398 36499726 PMC9738292

[38] Rebois RV. Establishment of gonadotropin-responsive murine leydig tumor cell line. J Cell Biol. (1982) 94:70−6. doi: 10.1083/jcb.94.1.70 6288740 PMC2112190

[39] Paradiso E Lazzaretti C Sperduti S Melli B Trenti T Tagliavini S. (Akt) blockade inhibits LH/hCG-mediated 17,20-lyase, but not 17α-hydroxylase activity of Cyp17a1 in mouse Leydig cell steroidogenesis. Cell Signal. (2023) 111:110872. doi: 10.1016/j.cellsig.2023.110872 37640196

[40] Luan Z Wei Y Huo X Sun X Zhang C Ming W. Pregnane X receptor (PXR) protects against cisplatin-induced acute kidney injury in mice. Biochim Biophys Acta Mol Basis Dis. (2021) 1867:165996. doi: 10.1016/j.bbadis.2020.165996 33127475

[41] Moore LB Parks DJ Jones SA Bledsoe RK Consler TG Stimmel JB. Orphan nuclear receptors constitutive androstane receptor and pregnane X receptor share xenobiotic and steroid ligands. J Biol Chem. (2000) 275:15122−7. doi: 10.1074/jbc.M001215200 10748001

[42] Zirkin BR Papadopoulos V. Leydig cells: formation, function, and regulation. Biol Reprod. (2018) 99:101−11. doi: 10.1093/biolre/ioy059 29566165 PMC6044347

[43] Liu X Sakai H Nishigori M Suyama K Nawaji T Ikeda S. Receptor-binding affinities of bisphenol A and its next-generation analogs for human nuclear receptors. Toxicol Appl Pharmacol. (2019) 377:114610. doi: 10.1016/j.taap.2019.114610 31195007

[44] Wyde ME Kirwan SE Zhang F Laughter A Hoffman HB Bartolucci-Page E. Di-n-butyl phthalate activates constitutive androstane receptor and pregnane X receptor and enhances the expression of steroid-metabolizing enzymes in the liver of rat fetuses. Toxicol Sci. (2005) 86:281−90. doi: 10.1093/toxsci/kfi204 15901914

[45] Lai TT Eken Y Wilson AK. Binding of per- and polyfluoroalkyl substances to the human pregnane X receptor. Environ Sci Technol. (2020) 54:15986−95. doi: 10.1021/acs.est.0c04651 33228354

[46] Zhang B Cheng Q Ou Z Lee JH Xu M Kochhar U. Pregnane X receptor as a therapeutic target to inhibit androgen activity. Endocrinology. (2010) 151:5721−9. doi: 10.1210/en.2010-0708 20962047 PMC2999492

[47] Manna PR Chandrala SP Jo Y Stocco DM. cAMP-independent signaling regulates steroidogenesis in mouse Leydig cells in the absence of StAR phosphorylation. J Mol Endocrinol. (2006) 37:81−95. doi: 10.1677/jme.1.02065 16901926

[48] Berkhout TA Simon HM Patel DD Bentzen C Niesor E Jackson B. The novel cholesterol-lowering drug SR-12813 inhibits cholesterol synthesis via an increased degradation of 3-hydroxy-3-methylglutaryl-coenzyme A reductase. J Biol Chem. (1996) 271:14376−82. doi: 10.1074/jbc.271.24.14376 8662919

[49] Moyle WR Jungas RL Greep RO. Metabolism of free and esterified cholesterol by Leydig-cell tumor mitochondria. Biochem J. (1973) 134:415−24. doi: 10.1042/bj1340415 16742800 PMC1177826

