# Supplementary material for: The Pregnane-X receptor regulates steroid synthesis in mouse Leydig cells
Source: Front Endocrinol (Lausanne). 2025 May 6;15:1430781. doi: 10.3389/fendo.2024.1430781 (PMC12088964; doi:10.3389/fendo.2024.1430781)
Supplement: Supplementary file 1 [file DataSheet1.docx]

# Supplementary Material


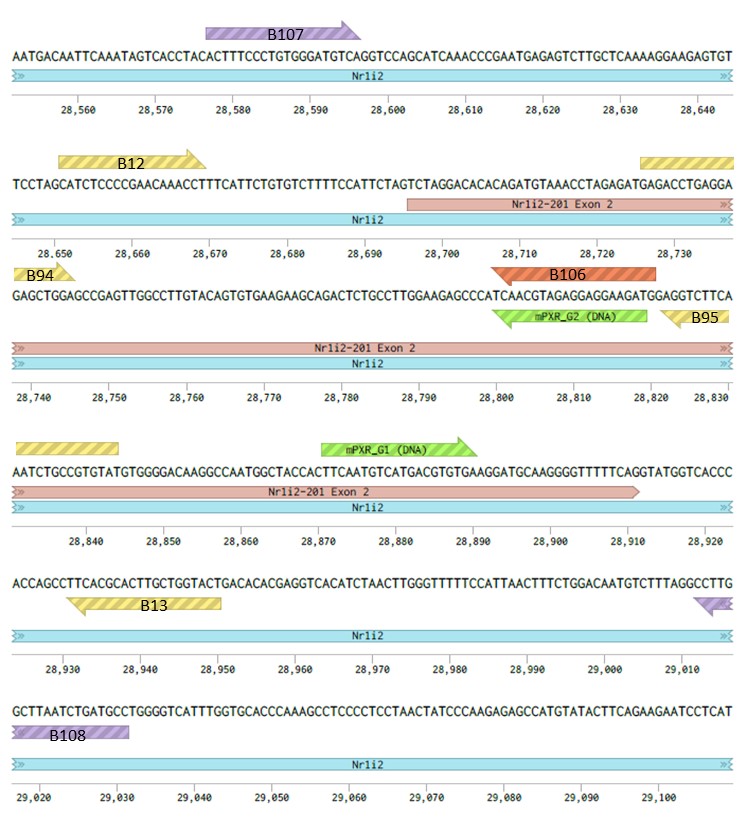


**Figure S1:** **Schemes for RNA guides and primers localisation used to generate and validate the *Pxr* Crispr/CAS9 edited MLTC-1 cells.** *Primers used in genotyping PCR in figures 2a and 2b: Primers B12 and B13 are located from each part of exon 2. Primers used for genotyping in figure 2c: B107 and B108 are located from each part of exon 2 and give a long fragment. Primers used for qPCR to amplify mouse Pxr in figure figure 2e: Primers B94 /B95 within the deleted zone PXR^KD^ and primers B94/were used to detect WT Pxr.*

*
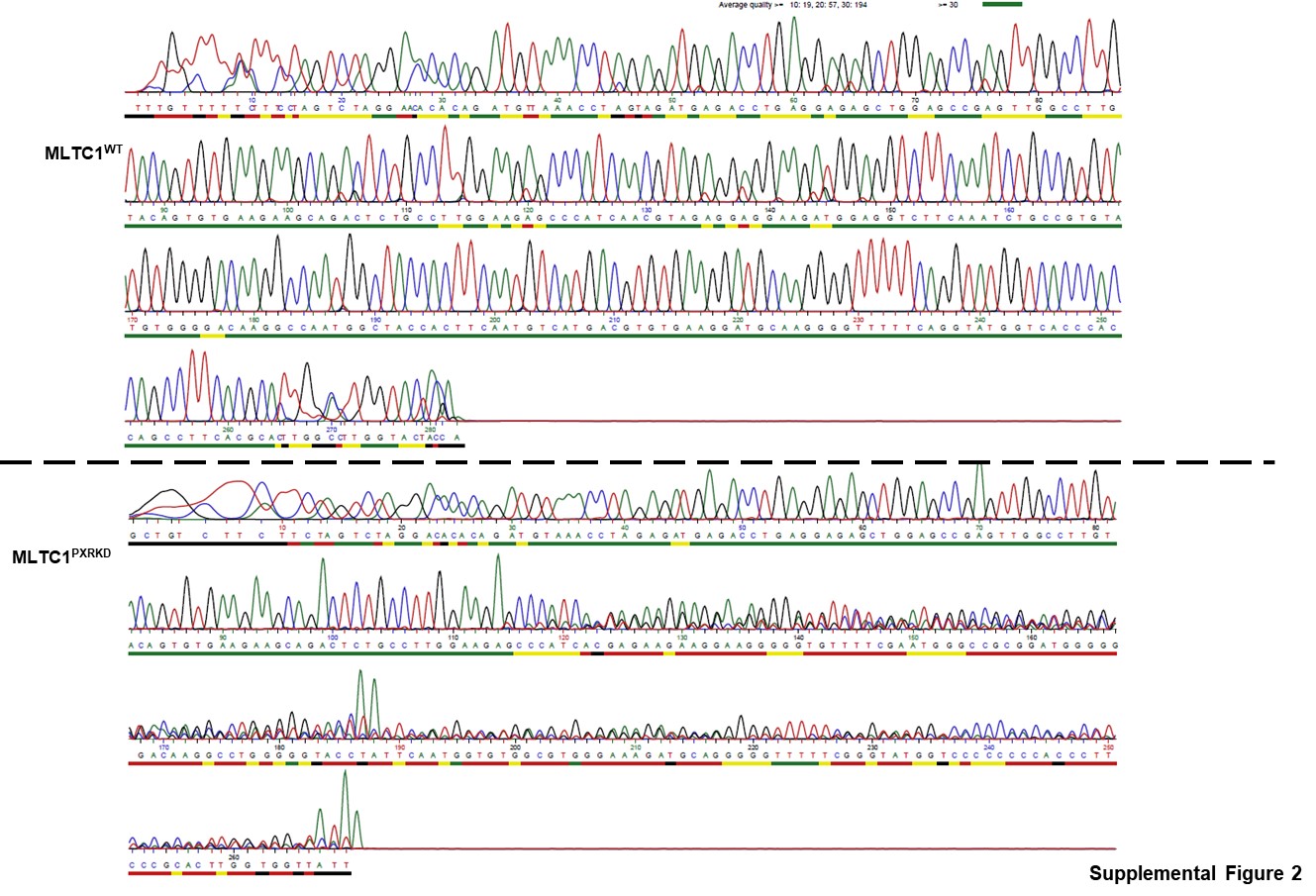
*

**Figure S2:** *Representative image of MLTC-1^WT^ and MLTC-1^PxrKD^ cells DNA sequencing data.*

**
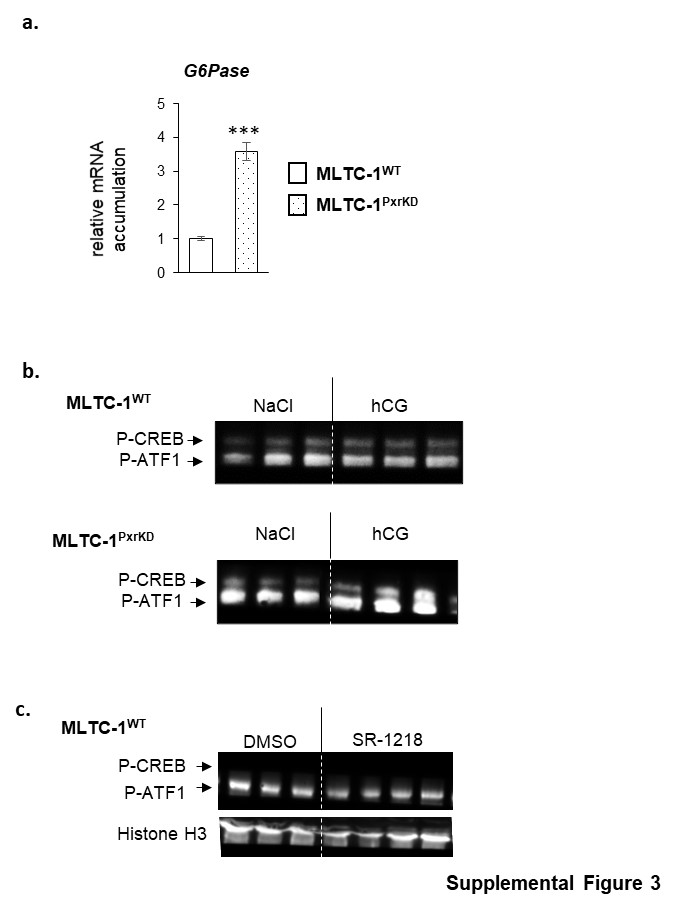
**

**Figure S3: (a)** mRNA accumulation of G6Pase normalized to b-actin in MLTC-1^WT^ and MLTC-1^PxrKD^ cells 24h after serum starvation. **(b)** Protein accumulation of P-CREB and HISTONE H3 in MLTC-1^WT^ and MLTC-1^PxrKD^ cells 24h after SR-1218 treatment.


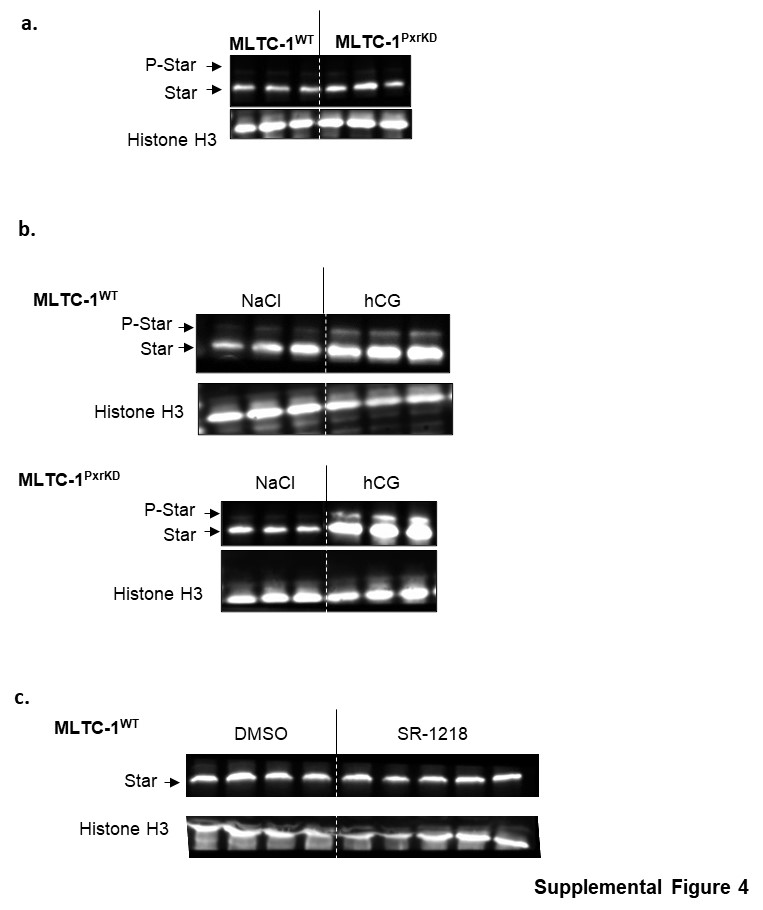


**Figure S4:** Protein accumulation of STAR and HISTONE H3 in MLTC-1^WT^ and MLTC-1^PxrKD^ cells 24h after SR-1218 treatment.

**Table S1:** *Sequences of RNA guides and primers used to generate and validate the Pxr Crispr/CAS9 edited MLTC-1 cells.*
